# Successful Outcome of a Corticodependent Henoch-Schönlein Purpura Adult with Rituximab

**DOI:** 10.1155/2014/619218

**Published:** 2014-04-01

**Authors:** Taylor Pindi Sala, Jean-Marie Michot, Renaud Snanoudj, Marion Dollat, Emmanuel Estève, Bernadette Marie, Yacine Taoufik, Jean-François Delfraissy, Thierry Lazure, Olivier Lambotte

**Affiliations:** ^1^Internal Medicine Department, Assistance Publique-Hôpitaux de Paris, Bicêtre Hospital, 94275 Le Kremlin-Bicêtre, France; ^2^Faculty of Medicine Paris-Sud, 94276 Le Kremlin-Bicêtre Cedex, France; ^3^Department of Nephrology and Adult Renal Transplantation, Assistance Publique-Hôpitaux de Paris, Necker Enfants-Malades Hospital, 75015 Paris, France; ^4^Hematology and Immunology Biological Department, Assistance Publique-Hôpitaux de Paris, Bicêtre Hospital, 94275 Le Kremlin-Bicêtre, France; ^5^Department of Pathology, Assistance Publique-Hôpitaux de Paris, Bicêtre Hospital, 94275 Le Kremlin-Bicêtre, France

## Abstract

Henoch-Schönlein purpura (HSP) is a systemic vasculitis involving small vessels with deposition of immunoglobulin A (IgA) complexes, usually affecting children. Compared with children, HSP in adults is more severe and frequently associated with cancer. We report the case of a 49-year-old woman with medical history of kidney transplantation for segmental glomerular hyalinosis. Eight years after the transplantation, while taking combined immunosuppressive therapy with tacrolimus and azathioprine indicated for the prevention against transplant rejection, she developed a Henoch-Schönlein purpura. Vasculitis involves skin and sciatic peroneal nerve and she received systemic corticosteroid treatment. Because of four relapses and corticosteroid dependence, the patient was treated with rituximab (two intravenous infusions of 1000 mg given two weeks apart). Successful outcome was observed along two years of follow-up. This new case of successful use of rituximab in HSP promotes more investigations of this treatment in clinical trials.

## 1. Background

Henoch-Schönlein purpura (HSP) is characterized by a leukocytoclastic vasculitis involving small vessels with deposition of immune IgA complexes [1]. HSP mainly affects children with an incidence around 15/100,000 per year [2] and is less frequent in adults [3]. Clinical symptoms include purpura, arthralgia, glomerulonephritis, and gastrointestinal and peripheral nerve involvement. Compared with children, clinical features and prognosis are different in adults: firstly the renal damage seems more frequent and serious, and then the frequency of associated cancers is higher [3, 4]. Cancers involved are bronchopulmonary, digestive, renal, and prostate. HSP prognosis is usually good, except in gastrointestinal and nephritis involvements. The treatment of HSP remains a matter of debate, both in adult and pediatric patients. Guidelines from pediatric experts do not support a systematic corticosteroid treatment [2, 5]. In adults the treatment usually consists in corticosteroids, eventually associated with an immunosuppressive treatment in cases of severe or relapsed forms [3, 4]. However, recent randomized studies failed to demonstrate a benefit to cyclophosphamide in addition to corticosteroids in severe glomerulonephritis forms in adults [6]. The B lymphocytes seem to be implicated in the pathogenesis of IgA nephropathy, a form of HSP limited to kidney [7, 8]. Consequently rituximab is a potential interesting targeted treatment and was already tested in three children [9] and two adult cases [10, 11] with severe HSP. We report here a new case of adult HSP successfully treated with rituximab.

## 2. Case Report

**Figure 1:**
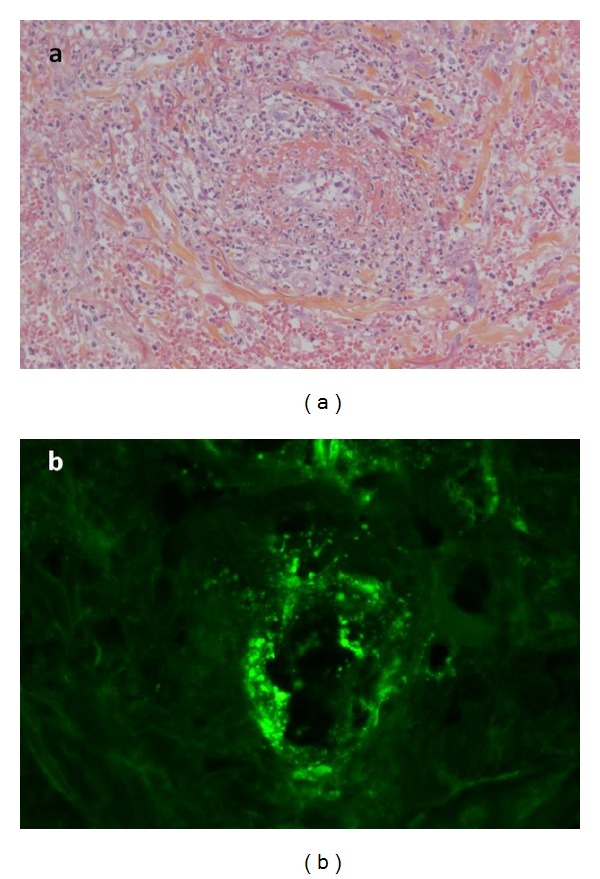
Skin biopsies of vasculitis lesions in lower limbs of the patient with refractory HSP (punch biopsy, 4 mm) were performed for routine histopathology and direct immunofluorescence. (a) Leukocytoclastic vasculitis: fibrin necrosis and infiltration of the blood vessel by neutrophils and conspicuous nuclear dust. Numerous extravasated blood cells (HES ×200). (b) IgA deposits in and around the vessel wall (direct immunofluorescence ×800).

A 49-year-old woman from mediterranean origin had eight-year history of end stage renal disease due to glomerulopathy, related to idiopathic focal segmental hyalinosis (immunofluorescence was negative in renal biopsy). The immunosuppressive therapy to prevent graft rejection included tacrolimus and azathioprine combination. Whereas the immunosuppressive treatment had not been recently modified, she developed an infiltrated and pruritic petechial purpura on the two lower limbs. Purpura was firstly localized in a perimalleolar area and then largely and rapidly extended on abdomen. She suffered from bilateral ankle arthralgia. She had no fever, no abdominal pain, and no rectal bleeding. She developed paresthesia and weakness in left lower limb in the sciatic peroneal nerve area. Two skin biopsies (punch biopsy, 4 mm) were performed for routine histopathology and direct immunofluorescence. Three micron sections cut from formalin-fixed, paraffin-embedded tissue were stained with hematoxylin, eosin, and saffron (HES). The frozen tissue sections were incubated with commercially prepared fluoresceinated antisera specific to human immunoglobulin's IgG, IgM, IgA, and complement three (C3). Lesions were characterized by an acute necrotizing inflammation of small vessels in the upper dermis with a dense perivascular and interstitial infiltrate of neutrophils, leukocytoclasia, and fibrin deposits into vascular wall and extravasated erythrocytes. Necrosis of the overlying epidermis led to intraepidermal vesiculation. Direct immunofluorescence revealed granular depositions of IgA and C3 in and around the walls of dermal small vessels (Figure 1). Laboratory blood tests showed normal hemoglobin (11.4 g/dL), leukocyte (5300/mm^3^), and platelet (251 G/L) counts. Serum creatinine level was stable at 107 mcmol/L and the patient remained in a chronic and stable moderate kidney disease. Serum albumin was 35 g/L and C-reactive protein 12 mg/L. HIV and hepatitis C serology were negative, and hepatitis B serology found a vaccine profile. The blood screenings for cryoglobulins, antinuclear antibodies, and antineutrophil cytoplasm antibodies were all negative. The complement values were within the normal ranges for C3: 0.78 mg/L (normal 0.66–1.25) and C4: 0.26 mg/L (normal 0.22–0.46) fractions. Serum immunoglobulin levels were increased for IgA (8.08 g/L; normal values 0.84–2.69) and normal for IgG (11.60 g/L; normal values 6.82–12.66) and IgM (1.98 g/L; normal values 0.66–1.87). The 24-hour proteinuria was moderately high at 0.26 grams, and urine cytology count was 260/mm^3^leukocytes and 40/mm^3^ red blood cells. Computed whole body tomodensitometry ruled out a tumor lesion.

**Figure 2:**
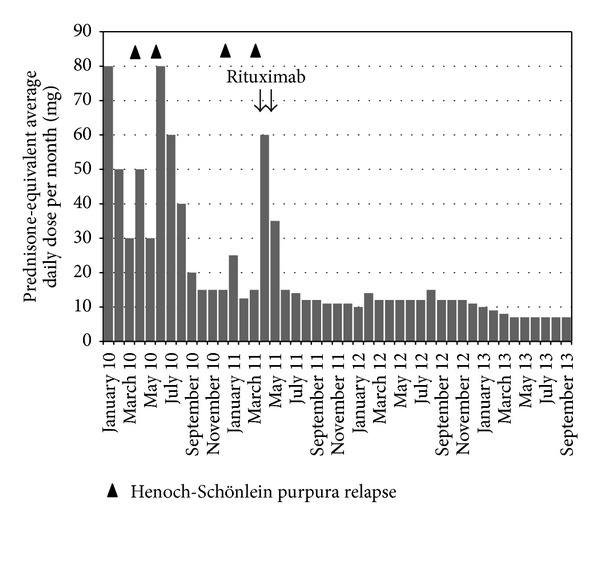
Corticosteroid daily doses received before and after rituximab treatment.

**Figure 3:**
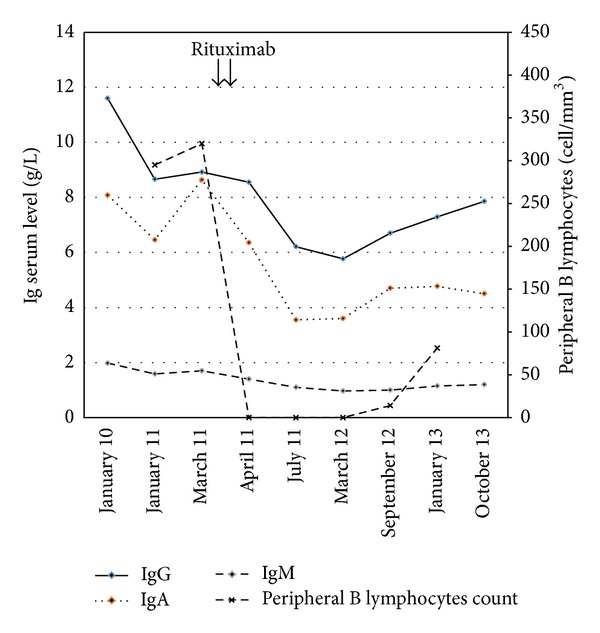
Serum immunoglobulin (Ig) levels and peripheral B lymphocytes count before and after rituximab treatment.

Henoch-Schönlein purpura (HSP) diagnosis was concluded with extended skin lesions and a peripheral nerve involvement (mononeuropathy in the sciatic peroneal nerve). Corticosteroid treatment was started using oral prednisone at one mg/kg daily dose. Tacrolimus and azathioprine (indicated for the prevention of transplant rejection) were continued without modification. After one month, the corticosteroid dosage was progressively tapered. Outcome within the first year was marked by four relapses of HSP (Figure 2), characterized by a large skin vasculitis and arthralgia flare, unresponsive to topical corticosteroids, and leading each time to increasing doses of systemic corticosteroids. Because of these repeated relapse, new investigations were performed, including a new whole body tomodensitometry which ruled out a cancer. In addition, a blood pharmacological assay of tacrolimus has verified the correct adherence of the patient to immunosuppressive treatment. The threshold of corticosteroid dependence was around 15 mg of total prednisone dose daily. The patient complained of a poor quality of life, due to the impact in everyday life practice of extensive pruritic skin lesions and weakness due to mononeuropathy, and asked to take another treatment. Because of the corticodependence and a significant impact of the disease in daily practical life of the patient, a new treatment was proposed and rituximab was suggested. The risk-benefit ratio was evaluated, especially considering the risk of infection, and the patient consented to receive this treatment. It was then decided to replace azathioprine with rituximab. Two intravenous infusions of rituximab 1000 mg were given two weeks apart. After the second rituximab infusion, azathioprine was gradually decreased and completely stopped after six months, in order to reduce the risk of infections related to immunosuppressive treatment. After rituximab, outcome was favorable without relapse. The patient had a gradual improvement in skin symptoms (vasculitis) and neurological (mononeuropathy), leading to a significant dose reduction of corticosteroids without relapse (Figure 2). We observed after rituximab therapy a progressive decrease in serum IgA level (Figure 3). The peripheral B lymphocytes cell count before rituximab was normal (320/mm^3^, 10% of total lymphocyte count) and fell to 0/mm^3^ one month after rituximab and reconstituting seventeen months later (Figure 3). Three months after the first infusion of rituximab, the patient had diarrhea related to* norovirus *infection, with a spontaneous favorable remission. There was no other infectious event until the date of writing this paper, with 30 months of follow-up.

## 3. Discussion

HSP treatment remains poorly codified, and corticosteroid therapy is discussed in children [2, 5]. In adults, treatment is based on local corticosteroid in limited skin involvement and on systemic corticosteroid in cases of visceral involvement [3, 4]. In refractory cases, the different immunosuppressive regimens proposed were variously azathioprine [12], cyclosporine A [13], or mycophenolate mofetil [14]. Efficacy of these immunosuppressive therapies remains largely uncertain because of the lack of controlled study. A recent prospective randomized study failed to demonstrate a benefit of cyclophosphamide in addition to corticosteroid in severe HSP glomerulonephritis in adults [6]. In front of the disappointment of these conventional immunosuppressive therapies, a new therapeutic approach with immunomodulating agents such as rituximab is raised.

Donnithorne et al. report three pediatric patients treated with rituximab for severe refractory chronic Henoch-Schönlein purpura [9]. All three patients respond to rituximab. Recently two adult patients received rituximab for severe and refractory HSP: complete skin and renal remission were reported in the thirst one [10], and renal and extrarenal manifestations eventually improved in the second one [11] after rituximab treatment. We here report a new case of adult patient with refractory HSP successfully treated by rituximab. We describe after rituximab a gradual improvement in skin symptoms (vasculitis) and neurological (mononeuropathy), leading to a dose reduction of corticosteroids without relapse. Regarding tolerance, we note the occurrence of gastrointestinal* norovirus* infection three months after rituximab, which resolved spontaneously. This infection has occurred when the serum globulin was low (IgG = 6 g/L). This infection and hypogammaglobulinemia may be related not only to rituximab treatment, but also to the other immunosuppressive treatment previously received.

In the present report, the link between the past history of initial nephropathy and eight years later is that the occurrence of HSP remains unclear. Retrospective analysis of the initial renal biopsy failed to demonstrate renal damages compatible with IgA nephropathy. The occurrence of IgA nephropathy after renal transplantation is much rarer than the classical recurrence of IgA nephropathy on graft, but it can happen exceptionally as described in two series [15, 16].

The cause of HSP remains to be determinate. The triggering factor, particularly* streptococcusβ*-hemolytic infection, concerns only a minority of patients [2]. A genetic susceptibility to develop the disease is likely [17]. Because of the involvement of IgA, HSP is expected to be a sick serum illness related to aberrant expression of autoantigen or exoantigen in the mucous membranes [17]. The IgA has a pivotal role in the pathophysiology of the disease, which is associated with a reduced IgA-immune complexes clearance [1]. The excess of these IgA-immune complexes leads to their deposition in various organs, particularly inducing glomerular mesangial damages in kidney. The glycosylation status of IgA is abnormally low in glomerulonephritis [18]. The hypogalactosylated IgA is recognized by anti-glycan IgG antibodies, leading to the immune complex and kidney damage [17]. Furthermore, enzymatic activity of IgA galactosylation was reduced in the B lymphocytes, while it was normal in both T lymphocytes and monocytes [7]. This data argues in favor of B lymphocyte implication in the HSP pathophysiology. Rituximab, eliminating B lymphocytes, may reduce the IgA-immune complexes during HSP flare and then reduce the disease activity. To investigate the potential benefit of rituximab in HSP or its limited kidney form (IgA nephropathy), it seems essential to target the patient's population suitable for this treatment in clinical trials. Indeed, these diseases are usually benign, and most of patients do not require immunosuppressive treatment. Candidates for rituximab treatment could be (a) patients with poor factor risks for progression to end stage renal disease, such as IgA nephropathy with severe inflammatory renal impairment, or (b) patients with severe HSP experiencing relapse, as a steroid sparing agent.
